# Carbon Dioxide-Derived Biodegradable and Cationic Polycarbonates as a New siRNA Carrier for Gene Therapy in Pancreatic Cancer

**DOI:** 10.3390/nano11092312

**Published:** 2021-09-06

**Authors:** Xinmeng Zhang, Zheng-Ian Lin, Jingyu Yang, Guan-Lin Liu, Zulu Hu, Haoqiang Huang, Xiang Li, Qiqi Liu, Mingze Ma, Zhourui Xu, Gaixia Xu, Ken-Tye Yong, Wei-Chung Tsai, Tzu-Hsien Tsai, Bao-Tsan Ko, Chih-Kuang Chen, Chengbin Yang

**Affiliations:** 1 Guangdong Key Laboratory for Biomedical Measurements and Ultrasound Imaging, School of Biomedical Engineering, Health Science Center, Shenzhen University, Shenzhen 518060, China; zhangxinmeng2019@email.szu.edu.cn (X.Z.); 2070246090@email.szu.edu.cn (J.Y.); 1910242069@email.szu.edu.cn (Z.H.); 1910242080@email.szu.edu.cn (H.H.); 2070246087@email.szu.edu.cn (X.L.); 2070246034@email.szu.edu.cn (Q.L.); mamz@szu.edu.cn (M.M.); xuzhouray@szu.edu.cn (Z.X.); xugaixia@szu.edu.cn (G.X.); 2 Polymeric Biomaterials Laboratory, Department of Materials and Optoelectronic Science, National Sun Yat-Sen University, Kaohsiung 80424, Taiwan; chengyen0624@gmail.com; 3 Department of Chemistry, National Chung Hsing University, Taichung 402, Taiwan; g106051074@mail.nchu.edu.tw; 4 Department of Biomedical Engineering, Southern University of Science and Technology, Shenzhen 518055, China; 5 School of Biomedical Engineering, The University of Sydney, Sydney, NSW 2006, Australia; ken.yong@sydney.edu.au; 6 The University of Sydney Nano Institute, The University of Sydney, Sydney, NSW 2006, Australia; 7 Division of Cardiology, Department of Internal Medicine, Kaohsiung Medical University Hospital, Kaohsiung 80708, Taiwan; azygo91@gmail.com (W.-C.T.); garytsaihsu@gmail.com (T.-H.T.)

**Keywords:** cationic polycarbonates, biodegradable, pancreatic cancer, siRNA delivery, K-ras

## Abstract

Pancreatic cancer is an aggressive malignancy associated with poor prognosis and a high tendency in developing infiltration and metastasis. K-ras mutation is a major genetic disorder in pancreatic cancer patient. RNAi-based therapies can be employed for combating pancreatic cancer by silencing K-ras gene expression. However, the clinical application of RNAi technology is appreciably limited by the lack of a proper siRNA delivery system. To tackle this hurdle, cationic poly (cyclohexene carbonate) s (CPCHCs) using widely sourced CO_2_ as the monomer are subtly synthesized via ring-opening copolymerization (ROCOP) and thiol-ene functionalization. The developed CPCHCs could effectively encapsulate therapeutic siRNA to form CPCHC/siRNA nanoplexes (NPs). Serving as a siRNA carrier, CPCHC possesses biodegradability, negligible cytotoxicity, and high transfection efficiency. In vitro study shows that CPCHCs are capable of effectively protecting siRNA from being degraded by RNase and promoting a sustained endosomal escape of siRNA. After treatment with CPCHC/siRNA NPs, the K-ras gene expression in both pancreatic cancer cell line (PANC-1 and MiaPaCa-2) are significantly down-regulated. Subsequently, the cell growth and migration are considerably inhibited, and the treated cells are induced into cell apoptotic program. These results demonstrate the promising potential of CPCHC-mediated siRNA therapies in pancreatic cancer treatment.

## 1. Introduction

Pancreatic cancer, the seventh leading cause of cancer death worldwide, has caused an estimated 432,242 deaths in 2018; it has a 5-y survival rate of only 9% and the highest incidence-to-mortality ratio among all solid tumors due to its high propensity to infiltrate and metastasize [1,2]. Moreover, the current clinical modalities of cancer treatment, such as chemotherapy and radiotherapy, have shown poor efficacy in improving its poor prognosis [3]. Accordingly, there is an urgently need in developing new therapeutic strategies aiming at overcoming this fatal disease. Recently, a number of studies revealed that pancreatic cancer was treated through the use of K-ras protooncogene inhibitors, which are capable of directly targeting mutant K-ras [4,5,6]. K-ras mutants present as dominant mutated oncogene isoforms in human cancer cells and K-ras mutants have been reported to be present in approximately 90% of pancreatic cancer cells [1,4,5,6,7]. K-ras is able to activate several downstream effector pathways (PI3K, RAF, and RalGEF), further resulting in tumor cell differentiation, growth, and anti-apoptosis [6]. Inhibition of the expression of K-ras directly correlated to the proliferation and the differentiation in cancer cells. Accordingly, the inhibition of K-ras is considered an effective methodology to address the dilemma in treating pancreatic cancer [8]. Unfortunately, all of the small-molecule inhibitors for K-ras have suffered from an unsatisfied result in clinical trials due to its well-known undruggable property [9]. So far, no therapeutic drugs directly targeting K-ras have been approved for clinical cancer treatment [10]. In this context, RNAi-based gene therapy has been considered a new hope to inhibit K-ras. Due to its efficacy in silencing specified mutated genes, RNAi therapy is thought a promising modality in treating intractable cancer diseases, such as pancreatic cancer. In August 2018, the first-ever RNAi-based drug, Alnylam’s Patisiran, was approved by the U.S. Food and Drug Administration (FDA) for treating transthyretin amyloidosis by inhibiting hepatic transthyretin production [11]. The siRNA molecules of patisiran were encapsulated by lipid particles coated with the apolipoprotein E (ApoE). After intravenous injection, this carrier specifically targets the liver through the binding of its ApoE receptors on hepatocytes. Then, the intracellular release of the loaded siRNA molecules is capable of silencing the TTR expression in hepatic cells. Such an approval of the siRNA-based new drug from FDA greatly encourages the enthusiasm of researchers on RNAi technology.

Having a negative charged surface, small interfering RNA (siRNA) is quite fragile due to its ready degradation by enzymes in serum. It is thus difficult for siRNA gene to directly penetrate into the cell membrane by itself [12]. Therefore, the development of a proper methodology for delivering siRNA to enter the cell membrane is of high importance on improving the therapeutic efficiency of siRNA-based gene therapies [13]. The use of nanocarriers has been thought one of the proper methods in delivering siRNA to target cells. In addition to delivering siRNA, suitable nanocarriers still need to overcome the biological barriers associated with siRNA delivery [14,15]. Nanocarriers used in gene delivery are divided into two categories: nonviral and viral vectors [16,17]. In recent years, due to the potential bio-risk associated with viral vectors, such as immune responses and insertional mutagenesis, nonviral vectors have gained a great deal of attention [18,19]. Cationic polymers have been reported to be the major developing type of nonviral vectors by virtue of the advantages of low toxicity, cost-effectiveness, facile production, and versatility [20,21,22]. Cationic polymers could form complex with anionic nucleic acids via electrostatic interaction [23,24]. The resulting complexation termed as polyplexes typically has the following favorable characters: (a) condensation ability for negatively charged nucleic acids; (b) protection of RNA from degradation; (c) overcome both intracellular and extracellular barriers; and (d) non-immunogenicity and high biocompatibility [18]. Toxicity and transfection efficiency generally are considered two key factors affecting the clinical applicability of siRNA carriers [14].

Although polyethylenimine (PEI)-based delivery systems have been demonstrated for their high transfection efficiency, their clinical uses are significantly hampered by their high toxicity, which typically results from the high molecular weight and non-degradability. Massive accumulation of non-degradable molecules in normal cells might adversely affect the metabolic activities [25]. Therefore, it is necessary to develop a biodegradable polymer with a reasonable charge density for the delivery of nucleic acids into the cell cytoplasm through the cell membrane, meanwhile avoiding the degradation in endosomes [26]. So far, biodegradable polymers used for gene delivery can be divided into synthetic polymer-based vectors and natural polymer-based vectors [20]. As compared with natural polymer-based vectors, synthetic polymer-based vectors are advantageous in the precise chemical structure control, high batch-to-batch uniformity and versatility in structural functionalization [27]. Commonly used synthetic polymer-based vectors include polyesters, polyurethanes, and polycarbonates. Of those polymers, polycarbonates feature for the favorable degradation mechanism, through which only non-acidic degradation residues that have no negative effects on normal tissues will generate [28]. Accordingly, aliphatic polycarbonates have been extensively employed for gene delivery [29]. However, those aliphatic polycarbonates, which generally resulted from ring-opening polymerization, are disadvantageous at their tedious synthesis procedure [30]. Moreover, with a T_g_ value ranging from 35–40 °C, the thermal stability of them is too low to satisfy rigorous sterilization treatment [31]. Recently, utilizing CO_2_ as the monomer, alicyclic polycarbonates such as poly(vinylcyclohexene carbonate) (PVCHC) can be synthesized via dinuclear nickel nitrophenolate-based catalysis [32]. Through the side chain functionalization treatment developed by our group, the hydrophilicity of such CO_2_-based alicyclic polycarbonates was largely enhanced. More importantly, those alicyclic polycarbonates exhibiting a Tg value higher than 115 °C, indicating the capability of standing sterilization treatment before the clinical uses. However, the prepared hydrophilic alicyclic polycarbonates have not been utilized as a gene delivery vector yet. With several unique biomedical merits, the newly developed alicyclic polycarbonates are considered to have a significant influence in gene delivery applications.

Several previous studies pointed out that direct siKras therapy has resulted in a significant inhibition of tumor proliferation and differentiation [1,2,7,33]. It was reported that utilizing siKras, a gene-targeted therapy, in two pancreatic cancer cell lines, PANC-1 and MiaPaCa-2, effectively inhibited tumor growth and obviously reduced the expression of the K-ras gene [20,34,35,36,37]. Furthermore, the angiogenic potential of both PANC-1 and MiaPaCa-2 cells was suppressed after siKras treatment [38]. A novel therapy strategy consisting of regulating TGF-β signaling via PEI as the carrier in advance and targeted delivering of siKras in using lipid-coated calcium phosphate biomimetic nanoparticles (siRNA Kras-LCPApoE3). This strategy was developed to aim at eliminating the stroma barrier and eventually enhancing the drug permeability into tumor sites [3]. All these previous findings clearly suggested that silencing the expression of K-ras via siRNA is an effective approach to overcome pancreatic cancer.

**Scheme 1:**
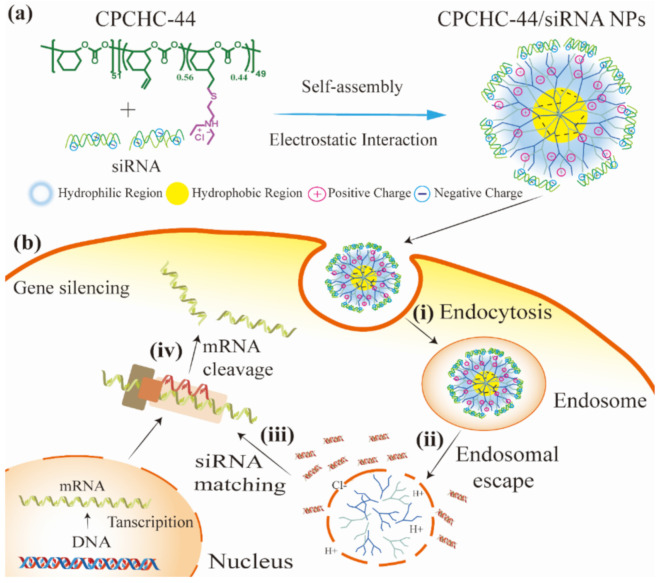
CPCHC-44 as siRNA carriers for pancreatic cancer treatment. (**a**) After a gently mixing, siRNAs were loaded into CPCHC-44 to form nanocomplexes via electrostatic interaction. (**b**) Conceptual cell uptake and transfection routes of CPCHC-44 as a nanocarrier for the siKras delivery for pancreatic cancer treatment. (**i**) Uptake of CPCHC-44/siRNA NPs through cell endocytosis. (**ii**) Rupture of endosomes and degradation of CPCHC-44 for the release of siRNAs into the cytosol. (**iii**) Unpacked siKras forms the RISC and matches with the targeting mRNA at the same time. (**iv**) Activate the RNAi mechanism and degrade the targeting mRNA.

In this study, a cationic and biodegradable polycarbonate, CPCHC-44, was synthesized and first utilized as a siRNA gene delivery carrier for combating pancreatic cancer. Selecting two pancreatic cancer cell lines, PANC-1 and MiaPaCa-2, as the study cells, the siRNA delivery capability and the K-ras silencing efficacy of CPCHC-44 can be systematically evaluated (Scheme 1). Through the copolymerization of CO_2_, cyclohexene oxide (CHO) and 4-vinyl-1,2-cyclohexene oxide (VCHO) by our developed catalyst and polymerization technique, a copolymer poly (cyclohexene carbonate)-*co*-poly (vinylcyclohexene carbonate) (PCHC-*co*-PVCHC) was successfully synthesized. Serving as a precursor polymer, PCHC-*co*-PVCHC was further transferred to CPCHCs by converting the vinyl functionalities on the polycarbonate backbone to tertiary amine groups through thiol-ene click modification. With an amine molar ratio of 44% relative to the PVCHC units, CPCHC-44 was prepared by controlling the reaction condition of the corresponding thiol-ene click functionalization.

Accordingly, in addition to its degradable carbonate main-chain, CPCHC-44 can be prepared by using CO_2_ as one of its constituent materials. Sequentially, self-assembly nanoplexes (NPs) were formed through the electrostatic interaction between positively charged CPCHC-44 and negatively charged siRNA. The CPCHC-44/siRNA NPs were then uptaken by cells through an endocytosis pathway [39]. After intracellular internalization, the proton-sponge mechanism via the tertiary amines of CPCHC-44 aided the escape of CPCHC-44/siRNA NPs from the endosomes into the cytoplasm. Next, the CPCHC-44/siRNA NPs unpacked the loaded siRNA through the degradation of cationic polymers in the cytosol, where the RNA-induced silencing complex (RISC)-mediated degradation of targeting K-ras mRNA was activated [22]. Our results showed that the CPCHC-44-mediated siRNA delivery significantly reduced the expression of K-ras. The K-ras gene silencing efficacy is comparable to that of a commercially transfection reagent, Lipofectamine3000 (Lipo3000). The anti-cancer performance of CPCHC-44/siKras was demonstrated by several cell proliferation and migration assays. The cytotoxicity evaluation further revealed that the excellent biocompatibility of CPCHC-44 especially at the necessary therapeutic dosage amounts. Due to the biomedical merits, CPCHC-44-mediaed gene therapies can be employed as an effective therapy strategy to treat pancreatic cancer in future.

## 2. Materials and Methods

### 2.1. Synthesis and Characterization of CPCHC-44

Utilizing a dinickel-based complex catalyst, ROCOP of CO_2_, CHO and VCHO was conducted for 4 h [32]. The resulting PCHC-*co*-PVCHC was characterized with 49% of pendant vinyl functionalities and *M*_n_^GPC^ of 19.7 kDa and served as the precursor polymer for CPCHC-44. In the synthesis of CPCHC-44, 100 mg of the precursor polymer was added in a 10 mL flask first. Sequentially, a quantitative amount of DEAET and DMPA was introduced to the flask, giving rise to the [allyl of PCHC-*co*-PVCHC]_0:_ [SH of DEAET]_0_: [DMPA]_0_ ratio of 1:2:0.4. Following the addition of the required agents, 5 mL of chloroform was used to dissolve them, followed by a freeze-pump-thaw procedure for three cycles. Subsequently, the resulting solution was exposed by a UV light (λ_max_ = 365 nm) for 30 min to perform the thiol-ene modification. After completing the modification, the unreacted reactants and catalysts were removed by dialysis using ethanol as the dialysis solvent, followed by drying the polymer in vacuum. The yield of CPCHC-44 was calculated to be 65–75%. ^1^H NMR (500 MHz, CDCl_3_, ppm) of CPCHC-44: *δ* 1.25–1.43 (br m, (CH_3_CH_2_)_2_N from the tertiary amine-modified units), 1.43–2.18 (br m, CH_2_CH_2_CH_2_CH_2_, CH_2_CH_2_CHCH_2_ from the CHC and VCHC units; CH_2_CH_2_CHCH_2_, CH_2_CH_2_CHCH_2,_ CHCH_2_CH_2_S from the tertiary amine-modified units), 2.34–2.49 (br m, CH_2_CH_2_CHCH_2_ from the VCHC units), 2.63–3.21 (br m, CH_2_CH_2_S, CH_2_SCH_2_CH_2_NCH_2_CH_2_ from the tertiary amine-modified units), 4.64–4.81 (br m, OCOCHCH from the CHC and VCHC units; OCOCHCH from the tertiary amine-modified units), 4.92–5.13 (br m, CHCHCH_2_ from the VCHC units), and 5.65–5.82 (br m, CHCHCH_2_ from the VCHC units). 

### 2.2. Agarose Gel Retardation Assay

First, siRNA and CPCHC-44 at different CPCHC-44/siRNA weight ratios were mixed by gently vortexed. The mixture was then incubated at room temperature for 1 h. Subsequently, the NPs were loaded on a 1.2% agarose gel and electrophoresed at 110 V for 9 min. The gel was then imaged under an ultraviolet (UV) transilluminator (FluorChem E, ProteinSimple, San Jose, CA, USA). The gray value was all analyzed by Image J.

### 2.3. The Formation and Characterization of CPCHC-44/siRNA NPs

The stability of the complex was verified by measurement with the hydrodynamic diameter and zeta potential (surface potential) values of the CPCHC-44/siRNA NPs in ultra-pure deionized water at room temperature with a 90-plus particle size analyzer (Zetasizer Nano-ZS90, Malvern). Moreover, the morphology of the CPCHC-44/siRNA NPs was observed on a HT7700 transmission electron microscope (TEM, Hitach, Ltd., Tokyo, Japan.).

### 2.4. Serum Enzymatic Protection Assay

To determine the stability of CPCHC-44/siRNA NPs in the presence of ribonuclease A, the CPCHC-44/siRNA NPs (the weight ratio of 16:1) incubated at a 0.5 μg/mL*2μL RNase A (Sigma Aldrich, St Louis, MO, USA) at room temperature for different incubation periods of 0, 6, 12, 18, 24 and 30 min. After culture, the solution was mixed with 1.0 wt% of SDS at 4 °C for 3 min and analyzed in Tris-acetate-EDTA Buffer (TAE, Thermo Fisher Scientific, New York, NY, USA) using 1.2% agarose gel electrophoresis. Then, the loading buffer was added and immediately loaded onto the agarose gel, followed by electrophoresis at 110 volts for 10 min. It was observed under an ultraviolet (UV) transilluminator. The remaining siRNAs were quantified according to the fluorescence intensity through the analysis from the software Image J. CPCHC-44 was then replaced by deionized water as the control group.

### 2.5. The Cytotoxicity Evaluation of CPCHC-44

Cell viability was measured by utilizing the Cell Counting Kit 8 (CCK8) assay (ab228554, abcam). Four different cell lines, namely, HUVEC, NIH/3T3, PANC-1, and MiaPaCa-2 cells were seeded in a 96-well plate at a density of 5 × 10^3^ cells/well and, leaving them to adhere for 24 h, then incubated with different concentrations of CPCHC-44/siRNA NPs for 24 h. CCK 8 (10 μL/well) in PBS was added and the cells were incubated for 2 h at 37 °C with 5% CO_2_. Absorbance was measured with a microplate reader (Bio-Tek) at a wavelength of 450 nm with a 5-min gentle shaking. Cell viability was calculated by normalizing the absorbance of the sample well to that of the control well, expressed as a percentage, and the viability of the untreated cells was specified as 100%.

### 2.6. Cell Culture

The human pancreatic cancer cells, PANC-1 (CRL-1469, American Type Culture Collection), MiaPaCa-2 (CRL-1420, American Type Culture Collection), the human endothelial cells, HUVEC (CRL-1730, American Type Culture Collection) and the fibroblast cell NIH/3T3 (CRL-1658, American Type Culture Collection)were maintained in culture with Dulbecco’s Modified Eagle’s Medium (DMEM, Gibco), supplemented with 10% fetal bovine serum (FBS, Gibco), penicillin (100 μg/mL, Gibco) and streptomycin (100 μg/mL, Gibco). Cells were cultured at 37 °C in a humidified atmosphere with 5% CO_2_.

### 2.7. siRNA Transfection

MiaPaCa-2 cells were seeded in a 6-well plate at a density of 1.5 × 10^6^ cells/well and left them to adhere at least for 24 h. The transfection begins as the confluence came to 50–70%. CPCHC-44 dispersion (1 mg/mL) was mixed with mutant K-ras siFAM (100 pM). The corresponding volumes for CPCHC-44/siFAM NPs are 20:10, 40:10, and 80:10 μL at a CPCHC-44/siFAM weight ratio of 16:1, 32:1, and 64:1, respectively. siFAM was gently mixed with CPCHC-44 and let stand at room temperature for 40 min. Before transfection, the culture medium was replaced with OPTI-MEM (Invitrogen), the above mentioned CPCHC-44/siFAM mixture was then added into the medium to reach a final volume of 1000 μL. After 4 h, the old medium-containing material was discarded, followed by washing it twice with phosphate-buffered solution (PBS). Sequentially, a DMEM medium (1000 μL) with 10% FBS was added to the medium. Free siFAM was also used in another parallel experiment. All groups except for blank, siRNA was kept at the same dosage level. A commercial transfection reagent Lipofectamine™ 3000 (Invitrogen) delivered siFAM was used as the positive control. The gene expression was monitored at 72 h post-transfection. For transfection efficiency examination, laser confocal imaging and flow cytometry assays were performed at a 4 h post-treatment. The custom synthesized K-ras siFAM was purchased from Shanghai GenePharma Co., Ltd. (Shanghai, China). The sense and antisense sequences of siKras were listed as follows. siKras for MiaPaCa-2 with sense: 5′-GUUGGAGCUUGUGGCGUAGUU-3′, antisense: 5′-CUACGCCACAAGCUCCACUU-3′ (siKras for MiaPaCa-2); siKras for PANC-1 with sense: 5′-FAM-GUUGGAGCUGAUGGCGUAGUU-3′, antisense: 5′-CUACGCCAUCAGCUCCAACUU-3′; scrambled siRNA(siNC) with sense: 5’-CGAAGUGUGUGUGUGUGGC-3, and antisense:’/5’-GCCACACACACACACUUCG-3’.

### 2.8. Confocal Imaging

A ZEISS LSM880 with AiryScan confocal microscope was used to capture in vitro fluorescence images. After 4 h of incubation with CPCHC-44/siRNA NPs at different CPCHC-44/siRNA weight ratios, the transfected cells were washed twice with PBS followed by fixation with 4% formaldehyde. Subsequently, the cells were stained with 4′, 6-diamidino-2-phenylindole (DAPI, Sigma) for nuclear counterstaining. The filter for the inverted microscope was set for DAPI (excitation at 405 nm and the emission was collected with a 450/50 nm band pass filter), FAM (excited with 488 nm laser and emission was collected with a band pass filter 525/50 nm), and Cy3 (excited with a 543 nm laser and the emission was collected with a 605/50 nm band pass filter). 

### 2.9. The Evaluation of Transfection Efficiency

For the flow cytometry experiments, post-transfected cells were washed twice with PBS and harvested by trypsinization (0.25%, without EDTA). The Cy3 served as the luminescent marker (filter set for ECD was applied) to determine the transfection efficiency quantitatively. For ECD channel, a set condition of 540 nm excitation and a 610/20 nm bandpass emission filter was used. The samples were analyzed on a flow cytometer (CytoFLEX, Beckman), and the resulting data were analyzed by the corresponding software CytExpert.

### 2.10. Gene Expression Assay

After the cells were transfected for 72 h, the transcription level of K-ras gene was investigated using quantitative real-time polymerase chain reaction (PCR) according to our previous experience [40]. In detail, the total RNA was extracted from PANC-1 and MiaPaCa-2 cells using a TRIzol reagent (Invitrogen) and quantified using a micro-spectrophotometer (Epoch2, BioTek Instruments). Total RNA (1 μg) was reversely transcribed to complementary deoxyribonucleic acid (DNA) using a reverse transcriptase kit from Promega in accordance with manufacturer’s instructions. The mRNA level of K-ras gene was measured by real-time PCR using a SYBR green dye (DBI-Bioscience 2143) in a QuantStudio 1 applied biosystem, followed by normalizing to the expression of β-actin. The primer sequences were as follows: Kras-F: 5′-AGAGTGCCTTGACGATAC-3′, 5′-ACCTGCTGTGTCGAGAAT-3′; β-actin F: 5′-GGTCATCACCATTGGCAATG-3′, and β-acting R 5′-TAGTTTCGTGGATGCCACAG-3′.

### 2.11. The Assessment of Cell Proliferation and Migration

The targeted anticancer effect of CPCHC-44/siRNA NPs on proliferation of PANC-1 and MiaPaCa-2 was determined by a CCK 8 assay as described above. The therapeutic effect on cell migration was determined using a wound-healing assay. In the assay, briefly, 1 × 10^4^ of PANC-1 cells were seeded in a 24-well plate followed by transfection with CPCHC-44/siRNA NPs at a CPCHC-44/siKras weight ratio of 16:1, 32:1, and 64:1 for 72 h, in which the cells almost achieved confluence. Then, a scratch wound was created on the surface containing the cells using a sterile pipette tip. The cells were continuously incubated for 72 h in a complete culture medium, and then the cells were imaged using an inverted fluorescence microscope. The rate of wound healing was determined by evaluating the area of migration window in the scratch wound at 0, 48 h, and 72 h (0 h is the time of scratching). Image J software was used to analyze the migration ratio.

### 2.12. Analysis of Apoptosis

The apoptosis rate of the transfected cells was determined using a FITC Annexin V Apoptosis Detection Kit I (BD Pharmingen). According to the kit assay’s protocol, 1 × 10^6^ PANC-1 and MiaPaCa-2 cells were seeded in a 6-well plate, followed by transfection with CPCHC-44/siKras NPs of all the formulations for 72 h. The dosage of siKras (100 pM, 10 μL/well) was kept constant in all group. The cells were then collected by trypsin (0.25%, without EDTA, Solarbio) and washed twice with cold PBS and suspended in a binding buffer. Then, flow cytometry (Beckman, CytoFLEX) was used to determine the number of apoptotic cells.

### 2.13. The Formation of 3D Tumor Spheroid Model

According to our previous paper [16,34], 1500 single viable MiaPaCa-2 cells were evenly distributed in 0.3% low melting point agarose gel (containing 10% FBS), and then the cells were seeded in 35 mm Petri dishes that were precoated with 0.6% low melting point agarose gel (containing 10% FBS). The cells were then incubated for 14 days to form colonies. After that, the cells were treated with siCy3 and CPCHC-44/siCy3. After incubation at 37 °C under 5% CO_2_ for 3 days, the resulting spheroids were gently rinsed with PBS and supplied with MEM (10% FBS, 100 µL). Then the permeation depth of formatted colonies were measured under a confocal laser scanning microscope (CLSM; λext = 532 nm, λobs = 550–650 nm).

### 2.14. Blood Test of CPCHC-44 NPs

The female BALB/c mice 5–6 weeks in age (around 20 g, *n* = 3) were obtained from the Medical Laboratory Animal Center of Guangdong Province, China. BALB/c mice were randomly divided into five groups and given an intravenous injection of either (1) control, (2) naked siKras (1 nmol siRNA dose per mouse), and (3) only CPCHC 44 (50 mg/kg) (4) CPCHC-44-scramble siRNA (1 nmol siRNA dose per mouse, 50 mg/kg CPCHC-44-equivalent dose), and (5) CPCHC-44/siKras (1 nmol siRNA dose per mouse, 50 mg/kg CPCHC-44-equivalent dose). The CPCHC-44 concentration in 50 mg/kg in each mouse was 10 times higher than that of the treatment mice during the in vivo study. At specified times after injection (0 to 16 days), the mice were weighed and evaluated by their healthy behavior. 15 days after the injection, the mice were sacrificed. A blood routine examination was performed using a routine blood test instrument (Mindray RJ-0C107223, China), and a biochemical marker analysis was performed using a blood biochemistry analyzer (Mindray BS-220, China). 

### 2.15. Xenograft Tumor Model Study

BALB/c nude female mice, which were 5 weeks in age, were purchased from the Medical Laboratory Animal Center of Guangdong Province, China, and raised in individually ventilated cages with sterile food, water, and bedding under a 12/12-h light/dark cycle. The mice were kept under normal observation for 14 days before carrying out the experiments. The animal experiments were performed according to the recommendations cited in the Guide for the Care and Use of Laboratory Animals of the Laboratory Animals Center of Shenzhen University. To generate a subcutaneous tumor xenograft, MiaPaCa-2 cell line were harvested at the exponential growth phase. One hundred microliters of the cell suspension (2 × 10^7^ cells per mL in PBS) was subcutaneously injected in the subaxillary region of each BALB/c mice. When the tumor grew to around 100 mm^3^, the mice were treated with 100 μL of PBS buffer, naked siKras, naked CPCHC 44, CPCHC 44-scramble siRNA and CPCHC 44/siKras via peritumoral injection at the abovementioned dosage (*n* = 6). Each mouse was treated once every three days, three times in total. To obtain the tumor growth curves, the formation of the tumor xenografts was monitored by measuring the tumor diameter using a caliper. The tumor volume was calculated using the equation L × W^2^/2, where L and W are the longest and shortest diameters of the tumor, respectively.

### 2.16. Statistical Analysis

All data were presented as mean ± SD. The results were compared by analysis of variance (ANOVA). All statistical calculations were performed with the SPSS 11.0 software package. When two comparisons were obtained, Student’s unpaired two-tailed *t*-test was used. A *p* value less than 0.01 was regarded as statistically significant.

## 3. Results and Discussion

### 3.1. Synthesis and Characterization of CPCHC-44

**Figure 1:**
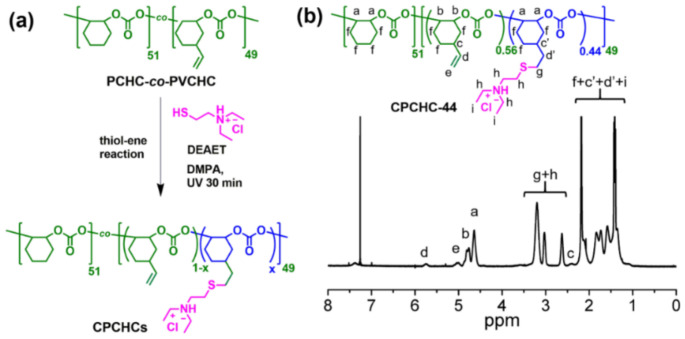
Synthesis and chemical structure of CPCHC44. (**a**) Synthetic route of CPCHCs. (**b**) ^1^H-NMR spectrum of CPCHC-44.

Resulting from CO_2_, a PCHC-*co*-PVCHC having 49% of pendant vinyl functionalities and *M*_n_^GPC^ of 19.7 kDa was synthesized via a ROCOP procedure through utilizing our developed dinickel-based complex catalyst in the ROCOP (Figure 1a) [32,41]. Following the synthesis, the vinyl functionalities of the PCHC-*co*-PVCHC were converted to tertiary amine groups in a controlled manner through a thiol-ene modification reaction. In the reaction, DEAET was reacted with the vinyl functionalities of the PCHC-*co*-PVCHC by using DMPA as the photo-initiator. With a [ene]_0_: [SH]_0_: [DMPA]_0_ molar ratio of 1:2:0.4 and a UV light treatemnt (λ_max_ = 365 nm) of 30 min, CPCHC-44 with an amine molar ratio of 44% relative to its polycarbonate backbone was synthesized. As shown in Figure 1b, the well-defined chemical structure of CPCHC-44 was analyzed through its ^1^H-NMR spectrum. The tertiary amine mol% of CPCHC-44 was calculated through the comparison of the resonance intensities of C*H*_2_SC*H*_2_C*H*_2_NC*H*_2_C*H*_2_ protons from tertiary-modified CHC units of CPCHC-44 at 2.63–3.21 ppm and the resonance intensities of C*H*C*H*O protons of cyclohexane rings from the three different units of CPCHC-44 at 4.64 and 4.76–4.81 ppm. Our developed thiol-ene functionalization technique not only can precisely control the charge density of CPCHCs but also enable the resulting CPCHCs with excellent water solubility when adjusting the amine mol% higher than 40%, such as CPCHC-44.

### 3.2. Cytotoxicity of CPCHC-44/siRNA NPs

The biocompatibility is one of the essential prerequisites of nanocarriers in delivering nucleic acid [32,33]. Thus, a MTT assay was performed to assess the cytotoxicity of CPCHC-44 toward cancer cell lines and normal cell lines, including human umbilical vein endothelial cells (HUVEC), normal embryonic fibroblast cell line (NIH/3T3), and two pancreatic cancer cell lines (PANC-1 and MiaPaCa-2). The results indicated that CPCHC-44 exhibited negligible cytotoxicity against these four cancer cell lines after 24 h. We observed that cell viability remained more than 85 ± 3.5% at a concentration up to 200 μg/mL (Figure S1). The sample concentrations used in the in vitro experiments for cancer cells generally ranged from 20–80 μg/mL. Accordingly, the use of CPCHC-44 at a common operating range would not cause any significant concerns about their cytotoxicity. As a siRNA carrier, the size, shape, and zeta potential of the resulting NPs are well-known to considerably affect the efficiency of their cellular uptake. As reported, due to their enhanced permeability and retention (EPR) effect, nanoparticles with an average particle size of 80–180 nm exhibited an enhanced aggregation at the tumor site [42]. In addition, such a particle size range is also beneficial for effectively reducing the clearance rate via the reticuloendothelial system and prolonging the blood circulation time in vivo [43,44]. The routine blood test and blood biochemical examination were taken to evaluate the biosafety of CPCHC-44 in vivo. Because the size of CPCHC 44-based nanoparticles is similar to viruses and large antigen, their immunogenicity may activate immune system and induce an acute inflammatory response, which can be reflected by monitoring the changes of hematological factors (such as red and white blood cell count). Accordingly, a complete blood routine test was conducted to investigate the immune response to CPCHC 44-based nanoparticles. As compared with the control group (PBS), CPCHC 44-based experimental groups including CPCHC-44, CPCHC-44/siNC, and CPCHC-44/siKras showed no distinct variation in the 16 hematological indicators (Figure S2). Similarly, the results from a serum biochemistry assay clearly suggested that the minimal interference of CPCHC-44-based experimental groups in metabolism activities of major organs (kidney and liver) (Figure S3). Besides, the body weight and physical behavior of the BALB/c mice were monitored during a 16-days observation period. No changes in drinking, eating, and physical behavior were observed between the CPCHC 44-treated and control mice (Figure S4). 

### 3.3. Preparation and Characterization of CPCHC-44/siRNA NPs

**Figure 2:**
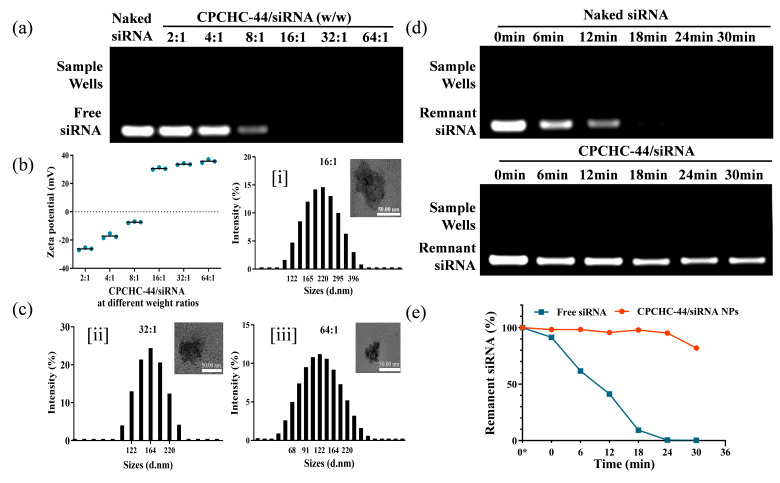
Characterization of CPCHC-44/siRNA NPs. (**a**) Electrophoretic mobility of CPCHC-44/siRNA nanocomplexes at different weight ratios. The dose of siRNA was fixed at 0.125 µg in each sample well. (**b**) Zeta potential for the nanoplexes at different CPCHC-44/siRNA weight ratios. (**c**) Particle sizes of CPCHC 44/siRNA nanocomplexes at CPCHC-44/siRNA weight ratio of 16:1[i], 32:1[ii], and 64:1[iii]. The inset indicates the TEM image of the corresponding CPCHC-44/siRNA NPs, scale bar = 50 nm. (**d**) Agarose gel electrophoresis results of remanent siRNA. The degradation of naked siRNA and CPCHC-44/siRNA (*w*/*w* = 16:1) after incubation with a RNase A-containing solution for a predetermined time to obtain the remnant siRNA. The fluorescence intensity corresponds to the remained siRNA amount was recorded on an agarose gel imaging system. (**e**) Quantification of remanent siRNA was performed through an ImageJ software. Results are presented as the mean ± SD, *n* = 3.

To investigate the optimal formulation of CPCHC-44 and siRNA when preparing CPCHC-44/siRNA NPs, CPCHC-44 was gently mixed with siRNA at different weight ratios of CPCHC-44 and siRNA. Sequentially, CPCHC-44/siRNA NPs were formed after 1 h through electrostatic interaction-induced self-assembly. It was observed that the amount of unbound free siRNA was steadily reduced with an increased proportion of CPCHC-44 (Figure 2a). When the weight ratio of CPCHC-44 and siRNA was close to 16:1, CPCHC-44 almost retarded all the siRNA in the sample well indicating most of the original siRNA has been successfully complexed and loaded by CPCHC-44. 

Subsequently, the surface zeta potential and size distribution of CPCHC-44/siRNA NPs were analyzed by dynamic light scattering (DLS) at different weight ratios. Results indicated that the zeta potential of the CPCHC-44/siRNA NPs was enhanced with an increase in the weight ratio of CPCHC-44 and siRNA due to the positive charged nature of the amine groups from CPCHC-44 (Figure 2b). The zeta potential of the formulated CPCHC-44/siRNA NPs was shown to be reversed from −26.23 ± 0.85 mV (weight ratio at 2:1) to +35.76 ± 1.16 mV (weight ratio 64:1). Such a change on the zeta potential of NPs was considered to facilitate the loaded siRNA to cross the negative charged cell membrane into cytoplasm [16]. NPs at CPCHC-44/siRNA weight ratios of 16:1, 32:1, and 64:1, were measured to have an average diameter of 220, 164, and 122 nm, respectively. An increase in the weight ratio of CPCHC-44/siRNA led to a decrease in the average NP size. This is because that a higher used amount of CPCHC-44 enhanced the complexing ability to siRNA, further resulting in a smaller size of the resulting NPs. TEM images clear observed the formation of CPCHC-44/siRNA NPs, which exhibited an irregular shape. At CPCHC-44/siRNA weight ratios of 16:1, 32:1, 64:1, the TEM result showed that the NPs has a particle size of 100, 80, and 50 nm (Figure 2c). The particle size of CPCHC-44/siRNA NPs observed from the TEM result consists very well with that from the DLS result. The ability of protecting siRNA from degradation by nucleases is an important indicator to evaluate the delivery performance of CPCHC-44. Accordingly, naked siRNA and CPCHC-44/siRNA NPs at the weight ratio of 16:1 were incubated at a high concentration RNase A solution for a predetermined time, followed by the separation of loaded siRNA from CPCHC-44/siRNA NPs using sodium dodecyl sulfate (SDS). The degradation level of siRNA was quantitatively analyzed by an agarose gel electrophoresis assay (Figure 2d). Results indicated that naked siRNA was notably degraded by RNase A (Figure 2e). In contrast, there was no apparent degradation of the siRNA under the protection of CPCHC-44. These results clearly indicated that CPCHC-44 is capable of effectively protecting siRNA from the degradation by RNase.

### 3.4. Intracellular Uptake and Transfection Efficiency of siRNA

**Figure 3:**
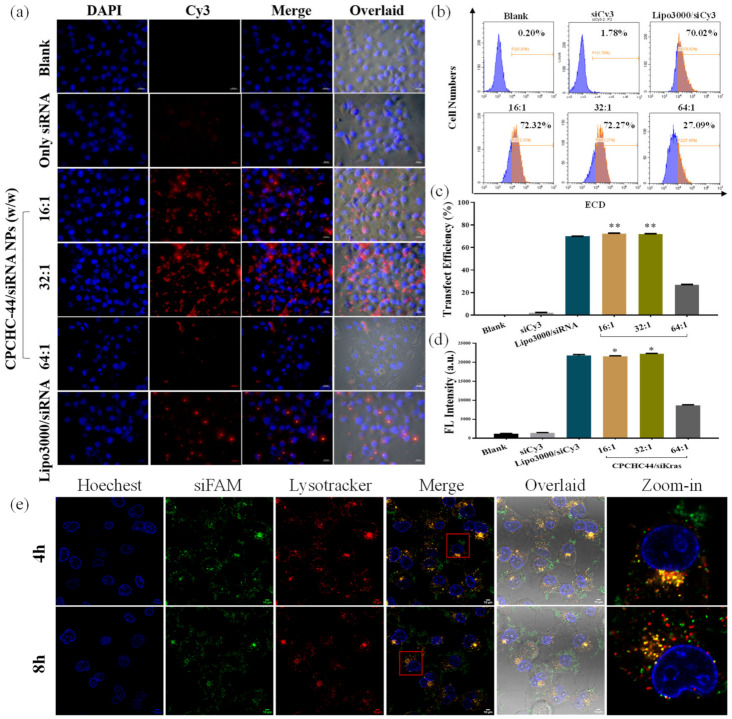
Fluorescence microscopy images of (**a**) PANC-1 cells treated with the tested formulations with the Lipo3000 as the positive control. Cell nuclei were stained with DAPI (pseudocolored blue), whereas the Cy3 signal is appeared as a red color. (**b**) Flow cytometry results for evaluating the siRNA delivery efficiencies of the tested formulations. The cell histograms were obtained from flow cytometry. (**c**) Transfection efficiencies and (**d**) average fluorescence intensities of the tested formulations. Both quantities were analyzed by flow cytometry. Values are mean ± SD, *n* = 3; Scale bar = 10 µm. (**e**) Confocal images revealed the cell uptake process of CPCHC-44/siFAM at different time intervals. The weight ratio of CPCHC-44/siFAM was fixed at 32:1, and the concentration of CPCHC-44 was 40 µg/mL. Cell endo-lysosomes labeled with LysoTracker Red exhibited a red fluorescence color, CPCHC-44/siFAM labeled with FAM exhibited a green fluorescence color, and cell nuclei labeled with Hoechst 33342 exhibited a blue fluorescence color. Scale bar = 10 µm. (*, *p* < 0.05; **, *p* < 0.01).

After the preparation of CPCHC-44/siRNA NPs, the siRNA delivery efficiency of CPCHC-44 was evaluated in vitro. In this context, the siRNA concentration was fixed at 1.25 µg mL^−1^, while the concentrations of CPCHC-44 were controlled at 20, 40, and 80 µg mL^−1^, giving rise to CPCHC-44/siRNA weight ratios of 16:1, 32:1, and 64:1. After co-incubation CPCHC-44/siCy3 and PANC-1 cancer cells for 5 h, the intracellular distribution of Cy3-labeled siRNA (siCy3) was observed under an inverted fluorescence microscope (Figure 3a). Non-treated (blank) and naked siCy3 were used as the negative controls, while the commercialized siRNA transfection reagent, Lipo3000, served as a siRNA carrier and the positive control. Similar to the blank group, no apparent Cy3 fluorescence signals were observed from the cells treated with naked siCy3. In contrast, significant intracellular Cy3 (red) fluorescence were observed in CPCHC-44/siCy3 NPs-treated groups, especially for the groups with a CPCHC-44/siCy3 weight ratio of 16:1 and 32:1. Moreover, the flow cytometry analysis was performed to quantify the transfection efficiency of siRNAs by CPCHC-44 (Figure 3b). The strong Cy3 fluorescence intensities in the CPCHC-44/siCy3 NPs- and Lipo3000/siCy3-treated groups were observed. More importantly, the PANC-1 cells treated with CPCHC-44/siCy3 NPs at 16:1 and 32:1 CPCHC-44/siCy3 weight ratios showed considerable number of Cy3-positive cells, in which the transfection efficiency was demonstrated to reach 72.68% in the group of the weight ratio of 16:1 (Figure 3c). Such a transfection efficiency was even slightly better than that achieved by the Lipo3000 (70.02%). Meanwhile, the transfection efficiencies of all the tested samples were consistent with their corresponding Cy3 fluorescence intensities from the confocal imaging results (Figure 3d). Taken together, these results clearly suggested that CPCHC-44 can be employed as an efficient gene delivery carrier for intracellular siRNA delivery in pancreatic cancer cells.

### 3.5. Endosomal Escape of siRNA Assisted by CPCHC-44

In addition to successfully shielding and delivering siRNA across the cell membrane, the capability of CPCHC-44 in overcoming intracellular barriers is also a crucial factor affecting the RNAi efficiency. Escaping from endosomal compartments into cytosol is considered one of the most challenging barriers for therapeutic siRNA to perform their transfection tasks [12,36]. Following penetrating into the cell membrane, siRNA wound enters the endosome in advance. Without the escaping from the endosome to the cytosol, siRNA will be transferred to the lysosome after the maturation of endosomal vesicles. Once moving to the lysosome, siRNA will be fast decomposed by lysosomal hydrolases, thereby causing a low efficiency in the subsequent RISC processing. Accordingly, the endosomal escaping efficiency of siRNA is a key factor affecting its transfection efficiency in treated cells [45]. To investigate the ability of CPCHC-44 in assisting the endo-lysosomal escape of siRNA, the intracellular location of CPCHC-44/siRNA NPs at a weight ratio of 32:1 was real-time tracked in PANC-1 cells using a confocal fluorescence microscope (Figure 3e). Endosomes labeled with LysoTracker Red exhibited red fluorescence, the encapsulated siRNA by CPCHC-44 was labeled with FAM, exhibiting green fluorescence, and cell nuclei was stained with Hoechst 33342, exhibiting blue fluorescence. After co-incubation PANC-1 cells with CPCHC-44/siFAM NPs for 4 h, yellow fluorescence signal was produced by merging the green fluorescence (FAM) with the red fluorescence (LysoTracker Red). This indicated that most of the CPCHC-44/siFAM NPs were trapped in the endosomal compartments first. When the incubation time was prolonged to 8 h, a considerable number of green fluorescence dots distinguishing from red fluorescence were observed, further resulting in a steady reduction area of overlapping yellow fluorescence. Such a result indicated CPCHC-44 is capable of aiding siFAM in endosomal escape. This endosomal escape mechanism of CPCHC-44/siRNA NPs can be credited by its tertiary amine groups, which led to a proton-sponge effect in the endosome and the lysosome. The protonated amine groups in the acidic endosome microenvironment caused a significant inflow amount of H^+^ protons. Such an inflow resulted in an equal amount of the counter Cl^−^ ions, which then give rise to a swelling and a partial disruption of the endosome. Such disruption is considered a major factor enabling CPCHC-44/siRNA NPs to escape from the endosomal compartments [46,47].

### 3.6. The Inhibition Effect of Kras Gene Transcription

**Figure 4:**
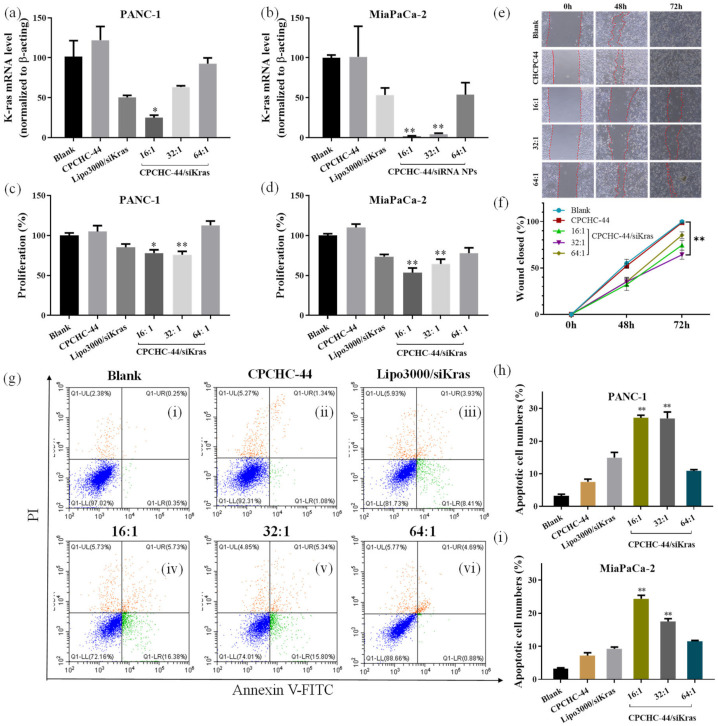
Anti-cancer efficacy of CPCHC-44/siRNA nanocomplex-mediated gene silencing in PANC-1 and MiaPaCa-2 cells. Determined by real-time PCR, expression levels of siKras were acquired in the treated (**a**) PANC-1 and (**b**) MiaPaCa-2. The gene expression of each tested formulation was normalized to that of β-actin. After treatment with the tested formulations, the proliferation of the treated (**c**) PANC-1 and (**d**) MiaPaCa-2 cells was determined using a MTT assay after a cultivation time for 72 h. Untreated cells were used as the control. (**e**,**f**) Wound healing assay results in PANC-1 cell treated with different concentrations of CPCHC-44/siRNA NPs. (**e**) Microscopic images for the wound healing status of the treated PANC-1 cells at 0 h, 48 h, and 72 h post-treatment. (**f**) Quantitative evaluation of the percentage of wound closed area through the estimation of the Image J software. The percent values were normalized on the basis of the initial wound window width. (**g**–**i**) Apoptosis assay results for PANC-1 and MiaPaCa-2 cells after the treatment of the tested formulations. After the treatment for 72 h, the cells were co-stained with annexin V and propidium iodide (PI), followed by a flow cytometry measurement. (**g**) Representative pictures of cells treated with (i) blank, (ii) CPCHC-44, (iii) Lipo3000/ siKras, and CPCHC-44/siKras NPs with various CPCHC-44/siKras weight ratios: (iv) 16:1, (v) 32:1, (vi) 64:1. (**h**,**i**) Apoptotic and dead cell percentages in the quadrants (Q1 + Q2 + Q4) for the cells treated with the tested formulations. Values are the mean ± SD, *n* = 3 (*, *p* < 0.05; **, *p* < 0.01).

After the siRNA was successfully delivered into the cytoplasm via CPCHC-44, the gene silencing effect of CPCHC-44/siRNA NPs was further investigated by real-time PCR on the PANC-1 (Figure 4a) and MiaPaCa-2 cells (Figure 4b). There was no significant difference in the gene expression between the groups of un-treated (blank) and CPCHC-44. The K-ras mRNA levels in both PANC-1 and MiaPaCa-2 cells were significantly inhibited after the cells were treated with CPCHC-44/siKras NPs and Lipo3000/siKras. The Lipo3000/siRNA treated group resulted in a consistent reduction in the mRNA level in both PANC-1 cell (increased by 50.42 ± 2.52%) and MiaPaCa-2 cells (increased by 53.39 ± 8.98%). The K-ras knockdown efficiencies of CPCHC-44/siKras at the weight ratios of 16:1 and 32:1 was 75 ± 3.17% and 36.6 ± 1.47%, (*p* < 0.01) in PANC-1 cells. Likewise, the knockdown efficiencies of CPCHC-44/siKras at the weight ratios of 16:1 and 32:1 were measured to be 98.2 ± 0.48% and 95.55 ± 1.67% (*p* < 0.01) in MiaPaCa-2 cells. Such a result has pointed out that the transfection efficiency of CPCHC-44 is superior to that of Lipo3000. Moreover, in both of the two cancer cell lines, CPCHC-44/siKras at the weight ratio of 64:1 showed an obvious much lower knockdown efficiency than that at the weight ratio of 16:1 and 32:1. It is considered that the increase in the weight ratio of CPCHC-44/siKras limits the unpacking ability of CPCHC-44. The poor unpacking of CPCHC-44/siKras at a high CPCHC-44/siKras weight ratio, such as 64:1, eventually led to a low K-ras knockdown efficiency. It is considered that CPCHC-44 with a significant amount of tertiary amines is able to induce “proton sponge effect” inside endosome/lysosome, resulting in the subsequent endosome/lysosome rupture and the unpacking of the loaded siRNA [15]. As compared with the commercial transfection agent Lipo3000, CPCHC-44 showed a stronger down-regulation level of K-ras mRNA expression. This is presumably due to the enhanced endosome/lysosome escaping and the proper siRNA unpacking ability from CPCHC-44 at its optimal CPCHC-44/siKras weight ratio.

### 3.7. Therapeutic Effects of CPCHC-44/siRNA NPs

After the treatment with different formulations for 72 h, PANC-1 (Figure 4c) and MiaPaCa-2 cells (Figure 4d) were evaluated respectively for the cell proliferation by MTT assay. The cell proliferation in the groups of CPCHC-44 and CPCHC-44/siNC NPs (siNC indicates the scrambled siRNA) show a similar growth trend to the blank group (Figure S5a,b). In contrast, when pancreatic cancer cells were treated with CPCHC-44/siRNA NPs at the weight ratios of 16:1, 32:1, and 64:1, treatments effectively resulted in inhibition of cell proliferation (46 ± 5.79%, 35 ± 5.83%, and 22 ± 6.58% in MiaPaCa-2 cells; 22 ± 3.93%, 25 ± 4.21% and 12 ± 5.39% in PANC-1 cells). Most importantly, it was noted that CPCHC-44/siRNA NPs treatment were more effective than the Lipo3000 commercial transfection agent in suppressing the cell proliferation in the MiaPaCa-2 cell (26 ± 2.68%) or PANC-1 cell (15 ± 4.13%). For patients with pancreatic cancer, metastasis, a complex process that initiates the invasion of primary cancer cells into the adjacent tissues, is confirmed to be a primary cause of mortality of the patients suffering from cancer [15]. Accordingly, a cell migration assay was performed to investigate the therapeutic effect of CPCHC-44/siRNA NPs. In the assay, a scratch wound was intentionally prepared by drawing a trace on a monolayer culture of adherent PANC-1 cells using a pipette tip. The therapeutic effect of different formulations on cell migration was observed based on the wound closing area at a predetermined time. As shown in Figure 4e, in the blank and the CPCHC-44 groups, after 48 h, it was clearly observed a reduction in the width of the wound; the straight boundary of the wound became disorganized, and cells began to migrate into the open space between the wound edges. After 72 h, the wound was almost completely healed, and the boundary was illegible. In contrast, the wound-healing was significantly inhibited in the cells treated with the CPCHC-44/siRNA NPs. The area width was decreased much less at the weight ratio of 16:1 and 32:1 group as compared to that in the other groups. The healing process of all the tested samples was further quantified and represented as a normalized percentage in each group (Figure 4f). In particular, after 72 h, the delivery of siKras via the CPCHC-44/siRNA NPs at the weight ratios of 32:1 and 16:1 was observed to display inhibition ratios in the wound closing of 37.71 ± 4.85% and 25.41 ± 5.3% respectively, indicating the significant suppression effect on the healing process of pancreatic cancer cells. 

Next, the performance of CPCHC-44/siRNA NPs on facilitating cell apoptosis was investigated using a FITC-labeled annexin V and PI staining kit. This method is established on the basis of the mechanism that phosphotidylserine on the cell membrane will flip from the inner side of the cell membrane to the surface of the cell membrane at the early stage of apoptosis. Annexin V has a high affinity to phosphotidylserine, which is merely exposed to the external cellular environment in apoptotic cells. As such, it has been widely used as an early apoptosis indicator. In addition, PI, a dye for binding DNA, is unable to cross the intact cell membrane of normal cells or early apoptotic cells; however, PI can cross the cell membrane and stain the cell nucleus with a red fluorescence in middle and late apoptotic cells and dead cells. The apoptotic cell number of the tested samples thus can be evaluated through the mentioned staining kit with flow cytometry, MiaPaCa-2 cells in (Figure 4g) and PANC-1 cells in (Figure S6). Results showed that the apoptotic cell numbers the CPCHC-44 treated group (7.53 ± 0.84%) are comparable with that of the blank groups (3.25 ± 0.5%). Those apoptotic cell number values indicated a negligible cell apoptosis result, especially as compared with other tested groups (Figure 4h,i). In contrast, the apoptotic cell numbers among PANC-1 cells in the experimental groups of CPCHC-44/siKras NPs at the weight ratios of 16:1, 32:1, 64:1 and Lipo3000/siRNA were increased to 27.15 ± 0.78%, 26.96 ± 1.98%, 10.86 ± 0.424%, and 14.9 ± 0.61%, respectively. In MiaPaCa-2 cells, the apoptotic cell numbers in the experimental groups of CPCHC-44/siKras NPs at the weight ratios of 16:1, 32:1, 64:1, and Lipo3000/siRNA were 24.4 ± 1%, 17.53 ± 0.826%, 11.5 ± 0.256%, and 9.25 ± 0.54%, respectively. Those results suggested that CPCHC-44/siKras NPs at the CPCHC-44/siKras weight ratio of 16:1 was considered the most efficient therapeutic formulation in facilitating the apoptosis of pancreatic cancer cells. 

Moreover, CPCHC-44 has shown an improved therapeutic efficacy than a commercial transfection reagent Lipo3000 on the basis of the in vitro results regarding the proliferation, migration, and apoptosis in pancreatic cancer cells. According to the results from CPCHC-44/siKras NPs-treated pancreatic cancer cells, CPCHC-44-mediated siRNA therapies are capable of addressing various therapeutic dilemmas in pancreatic cancer. Moreover, CPCHC-44 also enables other therapeutic siRNA genes to efficiently perform the designed transfection task in other cancer cell lines. Xenograft tumor model was performed to evaluate the in vivo anti-cancer effect of CPCHC-44/siKras NPs. The designed experimental groups included PBS, siKras, CPCHC-44, CPCHC-44/siNC, and CPCHC-44/siKras. The results showed that no significant change was observed in the weight of the treated mice from all the experimental groups (Figure S7). As compared with the other groups such as PBS, siKras, CPCHC-44, and CPCHC/siNC, the tumor volume was significantly reduced in the CPCHC/siKras-treated group after the first administration (Figure S8). These results clearly demonstrated the application potential of CPCHC-44 as siRNA carrier for pancreatic cancer gene therapy.

### 3.8. The Penetration Ability of CPCHC-44 NPs on 3D Tumor Spheroid Model

**Figure 5:**
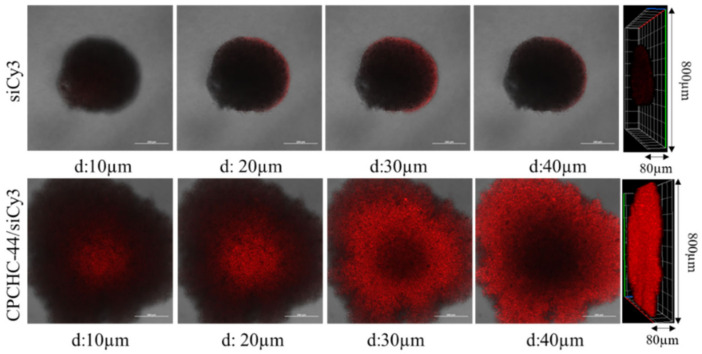
Confocal laser scanning microscopy mages of the 3D tumor spheroids incubated with the CPCHC-44/siCy3 NPs for 72 h. Scale bars: 200 μm.

To evaluate the penetration ability of CPCHC-44 NPs, the 3D-cultured MiaPaCa-2 spheroids were created in advance. MiaPaCa-2 cells were seeded between several agarose gels in a sandwich structure. The cells were then treated with siCy3 and CPCHC-44/siCy3 for 72 h when the diameter of the spheroids reached 500 μm. After treated with CPCHC-44/siCy3, the spheroids were observed by CLSM (confocal laser scanning microscopy). CLSM images showed that siCy3 via CPCHC-44/siCy3 has penetrated multiple layers of cells into the core of the 3D tumor spheroid in different cross sections (Figure 5). In contrast to the CPCHC-44/siCy3-treated group, the siCy3-treaed group only had the red fluorescence signal mainly distributing on the periphery of the tumor spheroids. These results demonstrated the deep tissue permeation ability of CPCHC-44/siCy3 in tumor spheroids, preliminary indicating the high potential of CPCHC-44 as siRNA carriers for in vivo applications.

## 4. Conclusions

In summary, utilizing CO_2_ as one of the constituent materials, CPCHC-44 was successfully synthesized by our developed ring-opening polymerization and thiol-ene modification techniques, which had biodegradability, controlled charge density, and insignificant cytotoxicity. More importantly, CPCHC-44, a new cationic alicyclic polycarbonate polymer, was first utilized as siRNA carriers for treating pancreatic cancer via silencing the K-ras gene expression. CPCHC-44/siKras NPs were prepared at various CPCHC-44 and siRNA weight ratios through electrostatic interaction. It was verified that CPCHC-44 is capable of protecting siRNA from be degraded by nucleases. At a specified CPCHC-44/siKras weight ratio, the loaded siKras payloads were effectively delivered into pancreatic cancer cells, followed by escaping from the endosomal compartments via the assistance of CPCHC-44. Eventually, CPCHC-44 unpacked the siKras payloads in the cytoplasm, enabling the siKras payloads to perform the required RNAi process. Due to the effective delivery of siKras via CPCHC-44, the activity of cell growth and migration in the treated PANC-1 and MiaPaCa-2 cells was significantly reduced, meanwhile the cell apoptosis level was remarkably enhanced. In particular, CPCHC-44/siKras NPs exhibited excellent therapeutic effect on MiaPaCa-2 cells. Besides, CPCHC-44 NPs demonstrated a deep tissue permeation ability on a 3D tumor spheroid model. Overall, by virtue of the biomedical merits of CPCHC-44, CPCHC-44/siRNA NPs can be further developed as new therapeutic strategy for pancreatic cancer treatment. In vivo studies of CPCHC-44/siRNA NPs in animal model, such as their biodistribution, immunotoxicity, and metabolism, are underway in our laboratory.

## Data Availability

The data presented in this study are available upon request from the corresponding author.

## Supplementary Materials

The following are available online at https://www.mdpi.com/article/10.3390/nano11092312/s1, Figure S1. The cytotoxicity of CPCHC-44 was evaluated by MTT assay on four cancer cell lines. The cells were incubated for 24 h with different concentrations of CPCHC-44. Blank cells were untreated. The results are represented as means ± SD, *n* = 5. Figure S2. Blood test results from the mice treated with CPCHC-44. Abbreviations: white blood cell count, WBC; lymphocyte count, Lymph; intermediate cell count, Mid; granulose count, Gran; red blood cell count, RBC; Hemoglobin, HGB; hematocrit, HCT; mean corpuscular volume, MCV; mean corpuscular hemoglobin, MCH; mean corpuscular hemoglobin concentration, MCHC; red cell distribution width, RDW; platelet count, PLT; mean platelet volume, MPV; platelet distribution width, PDW; plateletcrit, PCT. Figure S3. Blood biochemistry analysis for the mice treated with CPCHC-44. Abbreviations: alanine transaminase, ALT; aspartate transaminase, AST; albumin, ALB; alkaline phosphatase, ALP; blood glucose, GLU; triglyceride, TG; uric acid, UA; lactate dehydrogenase, LDH; creatine kinase, CK; total cholesterol, TC; high-density lipoprotein cholesterol, HDL-C; low-density lipoprotein cholesterol, LDL-C; direct bilirubin, D-BIL; gamma glutamyl transferase, γ-GT; α-Hydroxybutyrate Dehydrogrnase, α-HBDH. Figure S4. The weight curves of mice were monitored when conducting the in vivo toxicity assessment for CPCHC-44. Figure S5: After treatment with different formulation for 72 h, the proliferation of PANC-1 (a) and MiaPaCa-2 (b) cells were determined using MTT assay. Untreated cells were used as control. siNC means scrambled siRNA without any targeting gene. The results are represented as means ± SD, *n* = 3. Figure S6: Flow cytometric analysis of cell apoptosis in PANC-1 and MiaPaCa-2 cells upon 72 h post-treatment treatments. The apoptotic cells were co-stained with annexin V and propidium iodide (PI). (a) Representative dot-pots of cells treated with (i) blank, (ii) CPCHC-44, (iii) Lipo3000/siKras, and different weight ratios of CPCHC-44/siRNA NPs: (iv) 16:1, (v) 32:1, (vi) 64:1. Figure S7. The body weight change profiles of different CPCHC 44-based formulations treatment. Figure S8. Tumor growth curves of different groups after different CPCHC 44-based formulations treatment. Tumor volumes have been normalized to the initial sizes.

## References

[1] Christenson E.S., Jaffee E., Azad N.S. (2020). Current and emerging therapies for patients with advanced pancreatic ductal adeno-carcinoma: A bright future. Lancet Oncol..

[2] Yoshida T., Ohnami S., Aoki K. (2004). Development of gene therapy to target pancreatic cancer. Cancer Sci..

[3] Pei Y., Chen L., Huang Y., Wang J., Feng J., Xu M., Chen Y., Song Q., Jiang G., Gu X. (2019). Sequential targeting TGF-beta signaling and KRAS mutation increases therapeutic efficacy in pancreatic cancer. Small.

[4] Moore A.R., Rosenberg S.C., McCormick F., Malek S. (2020). RAS-targeted therapies: Is the undruggable drugged?. Nat. Rev. Drug Discov..

[5] Buscail L., Bournet B., Cordelier P. (2020). Role of oncogenic KRAS in the diagnosis, prognosis and treatment of pancreatic cancer. Nat. Rev. Gastroenterol. Hepatol..

[6] Liu P., Wang Y., Li X. (2019). Targeting the untargetable KRAS in cancer therapy. Acta Pharm. Sin. B.

[7] Aslan M., Shahbazi R., Ulubayram K., Ozpolat B. (2018). Targeted therapies for pancreatic cancer and hurdles ahead. Anticancer Res..

[8] Ryan M.B., Corcoran R.B. (2018). Therapeutic strategies to target RAS-mutant cancers. Nat. Rev. Clin. Oncol..

[9] Kessler D., Gmachl M., Mantoulidis A., Martin L.J., Zoephel A., Mayer M., Gollner A., Covini D., Fischer S., Gerstberger T. (2019). Drugging an undruggable pocket on KRAS. Proc. Natl. Acad. Sci. USA.

[10] Hamarsheh S.a., Groß O., Brummer T., Zeiser R. (2020). Immune modulatory effects of oncogenic KRAS in cancer. Nat. Commun..

[11] Titze-de-Almeida R., David C., Titze-de-Almeida S.S. (2017). The race of 10 synthetic rnai-based drugs to the pharmaceutical market. Pharm. Res..

[12] Kim B., Park J.-H., Sailor M.J. (2019). Rekindling RNAi therapy: Materials design requirements for in vivo sirna delivery. Adv. Mater..

[13] Yang C., Yang G., Ouyang Q., Kuang S., Song P., Xu G., Poenar D., Zhu G., Yong K., Wang Z.J.N.E. (2019). Nanowire-array-based gene electro-transfection system driven by human-motion operated triboelectric nanogenerator. Nano Energy.

[14] Saw P.E., Song E.W. (2020). siRNA therapeutics: A clinical reality. Sci. China Life Sci..

[15] Yang C., Chan K.K., Lin W.-J., Soehartono A.M., Lin G., Toh H., Yoon H.S., Chen C.-K., Yong K.-T. (2017). Biodegradable nanocarriers for small interfering ribonucleic acid (siRNA) co-delivery strategy increase the chemosensitivity of pancreatic cancer cells to gemcitabine. Nano Res..

[16] Yang C., Yin M., Xu G., Lin W.J., Chen J., Zhang Y., Feng T., Huang P., Chen C.K., Yong K.T. (2019). Biodegradable polymers as a noncoding miRNA nanocarrier for multiple targeting therapy of human hepatocellular carcinoma. Adv. Healthc. Mater..

[17] Setten R.L., Rossi J.J., Han S.P. (2019). The current state and future directions of RNAi-based therapeutics. Nat. Rev. Drug Discovery.

[18] Yin H., Kanasty R.L., Eltoukhy A.A., Vegas A.J., Dorkin J.R., Anderson D.G. (2014). Non-viral vectors for gene-based therapy. Nat. Rev. Genet..

[19] Thomas C.E., Ehrhardt A., Kay M.A. (2003). Progress and problems with the use of viral vectors for gene therapy. Nat. Rev. Genet..

[20] Chen C.K., Huang P.K., Law W.C., Chu C.H., Chen N.T., Lo L.W. (2020). Biodegradable polymers for gene-delivery applications. Int. J. Nanomed..

[21] Thomas T.J., Tajmir-Riahi H.A., Pillai C.K.S. (2019). Biodegradable polymers for gene delivery. Molecules.

[22] Hosseinkhani H., Domb A.J. (2019). Biodegradable polymers in gene-silencing technology. Polym. Adv. Technol..

[23] Yang C., Panwar N., Wang Y., Zhang B., Liu M., Toh H., Yoon H.S., Tjin S.C., Chong P.H., Law W.C. (2016). Biodegradable charged polyester-based vectors (BCPVs) as an efficient non-viral transfection nanoagent for gene knockdown of the BCR-ABL hybrid oncogene in a human chronic myeloid leukemia cell line. Nanoscale.

[24] Varkouhi A.K., Lammers T., Schiffelers R.M., van Steenbergen M.J., Hennink W.E., Storm G. (2011). Gene silencing activity of siRNA polyplexes based on biodegradable polymers. Eur. J. Pharm. Biopharm..

[25] Park T.G., Jeong J.H., Kim S.W. (2006). Current status of polymeric gene delivery systems. Adv. Drug Deliv. Rev..

[26] Jones C.H., Chen C.K., Chen M.F., Ravikrishnan A., Zhang H.G., Gollakota A., Chung T.C., Cheng C., Pfeifer B.A. (2015). PEGylated cationic polylactides for hybrid biosynthetic gene delivery. Mol. Pharmaceutics.

[27] Sabir M.I., Xu X.X., Li L. (2009). A review on biodegradable polymeric materials for bone tissue engineering applications. J. Mater. Sci..

[28] Zhang Z., Kuijer R., Bulstra S.K., Grijpma D.W., Feijen J. (2006). The in vivo and in vitro degradation behavior of poly(trimethylene carbonate). Biomaterials.

[29] Chen W., Meng F.H., Cheng R., Deng C., Feijen J., Zhong Z.Y. (2014). Advanced drug and gene delivery systems based on functional biodegradable polycarbonates and copolymers. J. Control. Release.

[30] Scharfenberg M., Hilf J., Frey H. (2018). Functional polycarbonates from carbon dioxide and tailored epoxide monomers: Degradable materials and their application potential. Adv. Funct. Mater..

[31] Li Y.J., Shimizu H. (2009). Compatibilization by homopolymer: Significant improvements in the modulus and tensile strength of PPC/PMMA blends by the addition of a small amount of PVAc. ACS Appl. Mater. Interfaces.

[32] Liu G.-L., Wu H.-W., Lin Z.-I., Liao M.-G., Su Y.-C., Chen C.-K., Ko B.-T. (2021). Synthesis of functional CO_2_-based polycarbonates via dinuclear nickel nitrophenolate-based catalysis for degradable surfactant and drug-loaded nanoparticle applications. Polym. Chem..

[33] Jiang H., Liu X., Knolhoff B.L., Hegde S., Lee K.B., Jiang H., Fields R.C., Pachter J.A., Lim K.H., DeNardo D.G. (2020). Development of resistance to FAK inhibition in pancreatic cancer is linked to stromal depletion. Gut.

[34] Yang C., Chan K.K., Xu G., Yin M., Lin G., Wang X., Lin W.J., Birowosuto M.D., Zeng S., Ogi T. (2019). Biodegradable polymer-coated multifunctional graphene quantum dots for light-triggered synergetic therapy of pancreatic cancer. ACS Appl. Mater. Interfaces.

[35] Lin G., Chen C.K., Yin F., Yang C., Tian J., Chen T., Xu G., He C., Lin M.C., Wang J. (2017). Biodegradable nanoparticles as siRNA carriers for in vivo gene silencing and pancreatic cancer therapy. J. Mater. Chem. B.

[36] Yang C., Hu R., Anderson T., Wang Y., Lin G., Law W.C., Lin W.J., Nguyen Q.T., Toh H.T., Yoon H.S. (2015). Biodegradable nanoparticle-mediated K-ras down regulation for pancreatic cancer gene therapy. J. Mater. Chem. B.

[37] Lin G., Hu R., Law W.C., Chen C.K., Wang Y., Li Chin H., Nguyen Q.T., Lai C.K., Yoon H.S., Wang X. (2013). Biodegradable nanocapsules as siRNA carriers for mutant K-Ras gene silencing of human pancreatic carcinoma cells. Small.

[38] Fleming J.B., Shen G.L., Holloway S.E., Davis M., Brekken R.A. (2005). Molecular consequences of silencing mutant K-ras in pancreatic cancer cells: Justification for K-ras-directed therapy. Mol. Cancer Res..

[39] Takei K., Haucke V. (2001). Clathrin-mediated endocytosis: Membrane factors pull the trigger. Trends Cell Biol..

[40] Yang C., Mo X., Lv J., Liu X., Yuan M., Dong M., Li L., Luo X., Fan X., Jin Z. (2012). Lipopolysaccharide enhances FcεRI-mediated mast cell degranulation by increasing Ca^2+^ entry through store-operated Ca^2+^ channels: Implications for lipopolysaccharide exacerbating allergic asthma. Exp. Physiol..

[41] Chang C.-H., Tsai C.-Y., Lin W.-J., Su Y.-C., Chuang H.-J., Liu W.-L., Chen C.-T., Chen C.-K., Ko B.-T. (2018). Alternating copolymerization of epoxides with carbon dioxide or cyclic anhydrides using bimetallic nickel and cobalt catalysts: Preparation of hydrophilic nanofibers from functionalized polyesters. Polymer.

[42] Chen C.K., Lin W.J., Hsia Y., Lo L.W. (2017). Synthesis of polylactide-based core-shell interface cross-linked micelles for anticancer drug delivery. Macromol. Biosci..

[43] Hickey J.W., Santos J.L., Williford J.M., Mao H.Q. (2015). Control of polymeric nanoparticle size to improve therapeutic delivery. J. Control. Release.

[44] Cabral H., Matsumoto Y., Mizuno K., Chen Q., Murakami M., Kimura M., Terada Y., Kano M.R., Miyazono K., Uesaka M. (2011). Accumulation of sub-100 nm polymeric micelles in poorly permeable tumours depends on size. Nat. Nanotechnol..

[45] Gao Y., Jia L., Wang Q., Hu H., Zhao X., Chen D., Qiao M. (2019). pH/Redox Dual-responsive polyplex with effective endosomal escape for codelivery of siRNA and doxorubicin against drug-resistant cancer cells. ACS Appl. Mater. Interfaces.

[46] Hosseinahli N., Aghapour M., Duijf P.H.G., Baradaran B. (2018). Treating cancer with microRNA replacement therapy: A literature review. J. Cell. Physiol..

[47] Wang H., Jiang Y., Peng H., Chen Y., Zhu P., Huang Y. (2015). Recent progress in microRNA delivery for cancer therapy by non-viral synthetic vectors. Adv. Drug Deliv. Rev..

