# Supplementary material for: Carbon Dioxide-Derived Biodegradable and Cationic Polycarbonates as a New siRNA Carrier for Gene Therapy in Pancreatic Cancer
Source: Nanomaterials (Basel). 2021 Sep 6;11(9):2312. doi: 10.3390/nano11092312 (PMC8472555; doi:10.3390/nano11092312)
Supplement: Supplementary file 1 [file nanomaterials-11-02312-s001.zip › nanomaterials-1309099-supplementary.pdf]

Supplementary Materials

Carbon Dioxide-Derived Biodegradable and Cationic Polycarbonates as a New siRNA Carrier for Gene Therapy in Pancreatic Cancer

Xinmeng Zhang [1], Zheng-Ian Lin [2], Jingyu Yang [1], Guan-Lin Liu [3], Zulu Hu [1], Haoqiang Huang [1], Xiang Li [1], Qiqi Liu [1], Mingze Ma [1,4], Zhourui Xu [1], Gaixia Xu [1], Ken-Tye Yong [5,6], Wei-Chung Tsai [7], Tzu-Hsien Tsai [7], Bao-Tsan Ko [3,*],
Chih-Kuang Chen [2,*] and Chengbin Yang [1,*]

[1] Guangdong Key Laboratory for Biomedical Measurements and Ultrasound Imaging, School of Biomedical Engineering, Health Science Center, Shenzhen University, Shenzhen 518060, China;
zhangxinmeng2019@email.szu.edu.cn (X.Z.); 2070246090@email.szu.edu.cn (J.Y.); 1910242069@email.szu.edu.cn (Z.H.); 1910242080@email.szu.edu.cn (H.H.); 2070246087@email.szu.edu.cn (X.L.); 2070246034@email.szu.edu.cn (Q.L.), mamz@szu.edu.cn (M.M.); xuzhouray@szu.edu.cn (Z.X.); xugaixia@szu.edu.cn (G.X.)

[2] Polymeric Biomaterials Laboratory, Department of Materials and Optoelectronic Science, National Sun
Yat-sen University, Kaohsiung 80424, Taiwan; chengyen0624@gmail.com

[3] Department of Chemistry, National Chung Hsing University, Taichung 402, Taiwan; g106051074@mail.nchu.edu.tw

[4] Department of Biomedical Engineering, Southern University of Science and Technology, Shenzhen 518055, China

[5] School of Biomedical Engineering, The University of Sydney, Sydney, NSW 2006, Australia; ken.yong@sydney.edu.au

[6] The University of Sydney Nano Institute, The University of Sydney, Sydney, NSW 2006, Australia.

[7] Division of Cardiology, Department of Internal Medicine, Kaohsiung Medical University Hospital, Kaohsiung 80708, Taiwan; azygo91@gmail.com (W.-C.T.); garytsaihsu@gmail.com (T.-H.T.)

* Correspondence: btko@dragon.nchu.edu.tw (B.-T.K.); chihkuan@mail.nsysu.edu.tw (C.-K.C); cbyang@szu.edu.cn (C.Y.); Tel.:+886-4-22840411 (ext. 715) (B.-T.K.); +886-7-5252000 (ext. 4060) (C.-K.C.); +86-0755-26932683 (C.Y.)

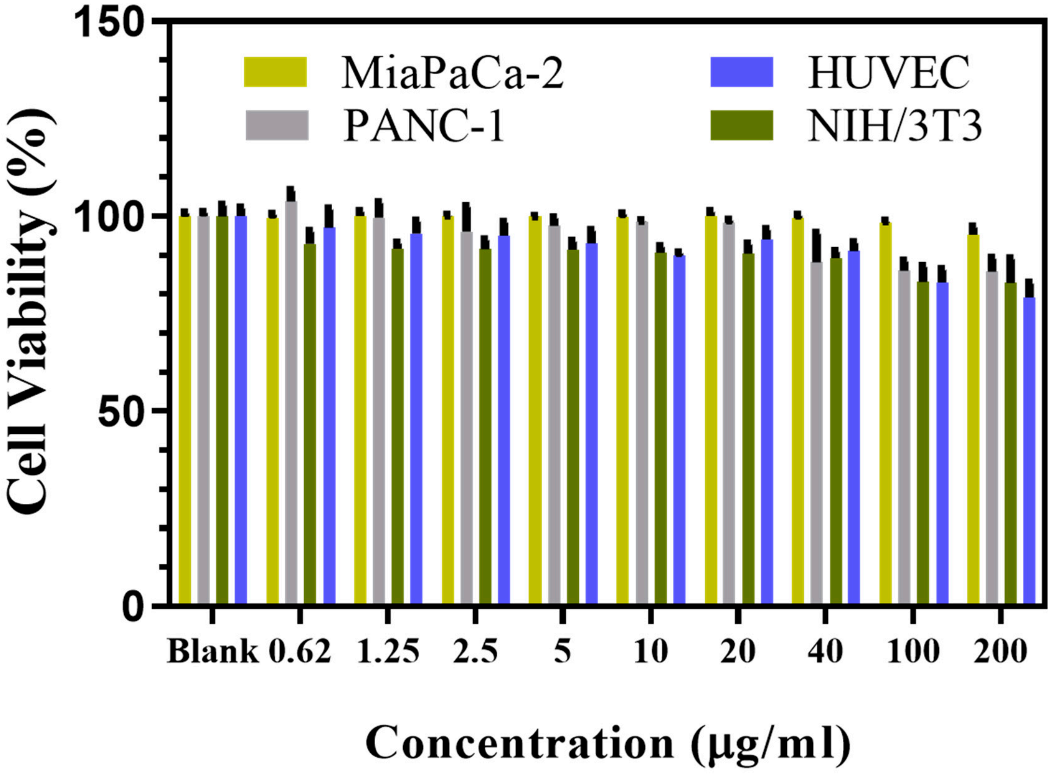

Figure S1. The cytotoxicity of CPCHC-44 was evaluated by MTT assay on four cancer cell lines. The cells were incubated for 24h with different concentrations of CPCHC-44. Blank cells were untreated. The results are represented as means ± SD, n = 5.

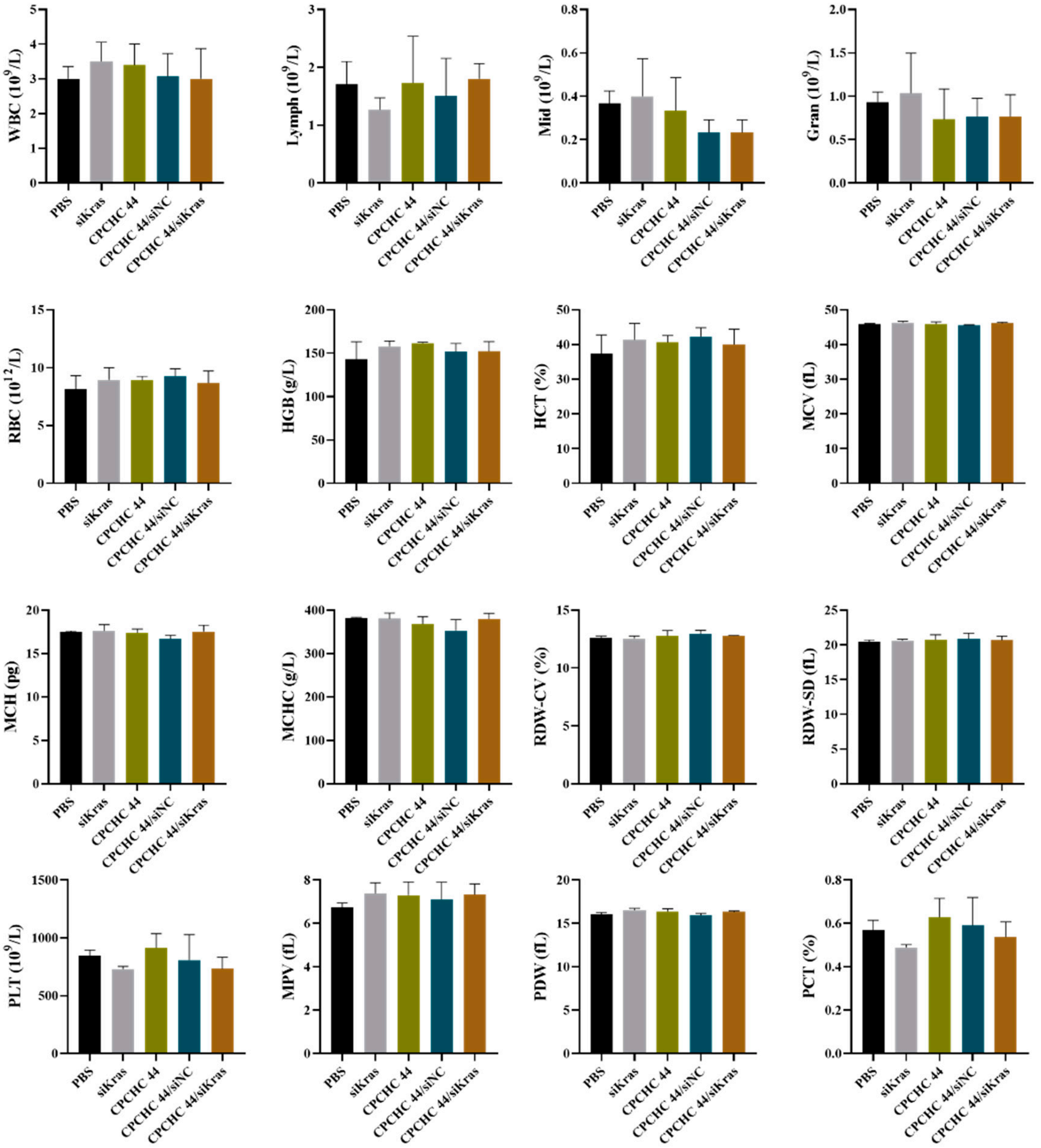

Figure S2. Blood test results from the mice treated with CPCHC-44. Abbreviations: white blood cell count, WBC; lymphocyte count, Lymph; intermediate cell count, Mid; granulose count, Gran; red blood cell count, RBC; Hemoglobin, HGB; hematocrit, HCT; mean corpuscular volume, MCV; mean corpuscular hemoglobin, MCH; mean corpuscular hemoglobin concentration, MCHC; red cell distribution width, RDW; platelet count, PLT; mean platelet volume, MPV; platelet distribution width, PDW; plateletcrit, PCT.

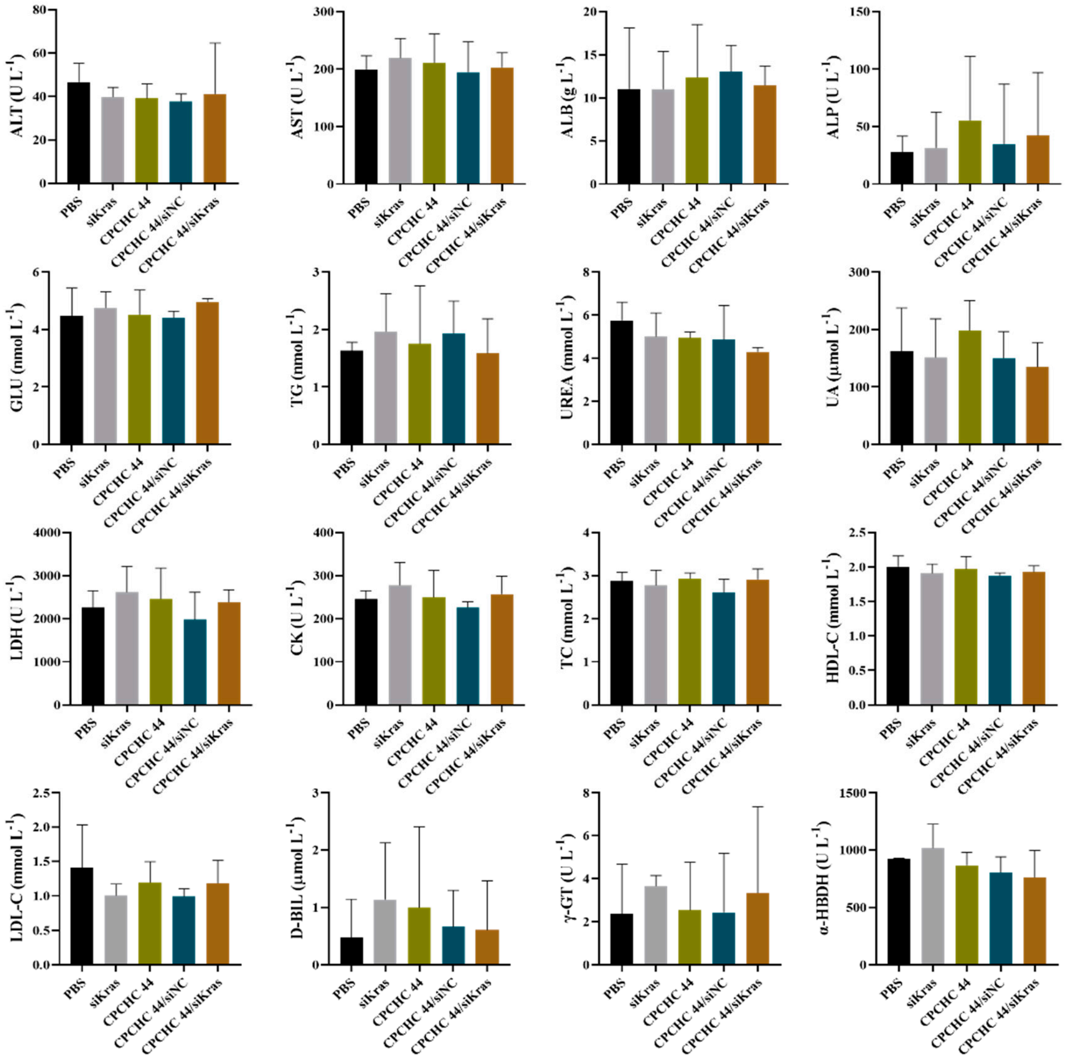

Figure S3. Blood biochemistry analysis for the mice treated with CPCHC-44. Abbreviations: alanine transaminase, ALT; aspartate transaminase, AST; albumin, ALB; alkaline phosphatase, ALP; blood glucose, GLU; triglyceride, TG; uric acid, UA; lactate dehydrogenase, LDH; creatine kinase, CK; total cholesterol, TC; high-density lipoprotein cholesterol, HDL-C; low-density lipoprotein cholesterol, LDL-C; direct bilirubin, D-BIL; gamma glutamyl transferase, γ-GT; α-Hydroxybutyrate Dehydrogrnase, α-HBDH.

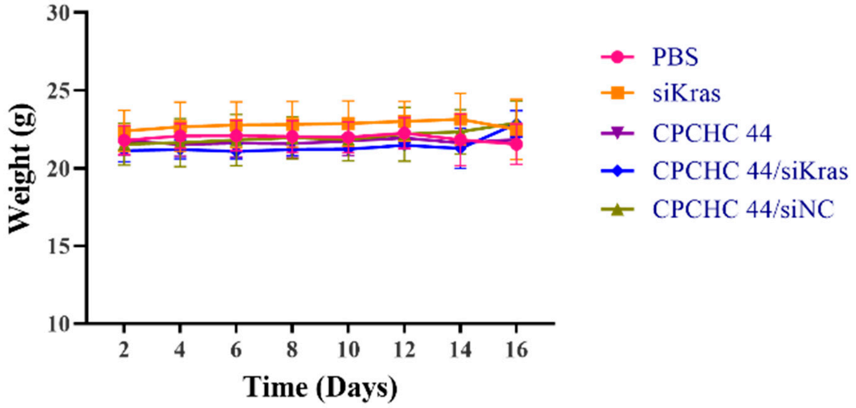

Figure S4. The weight curves of mice were monitored when conducting the *in vivo* toxicity assessment for CPCHC-44.

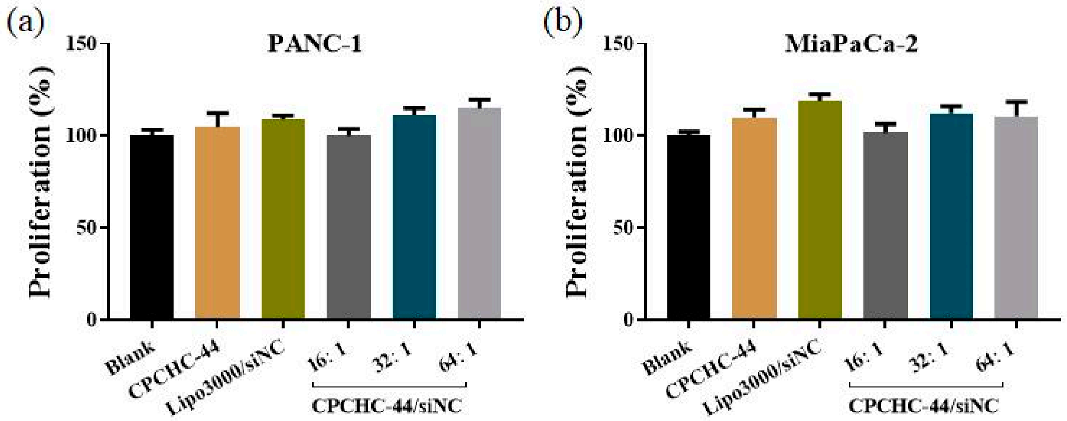

Figure S5. After treatment with different formulation for 72 h, the proliferation of PANC-1 (a) and MiaPaCa-2 (b) cells were determined using MTT assay. Untreated cells were used as control. siNC means scrambled siRNA without any targeting gene. The results are represented as means ± SD, n=3.

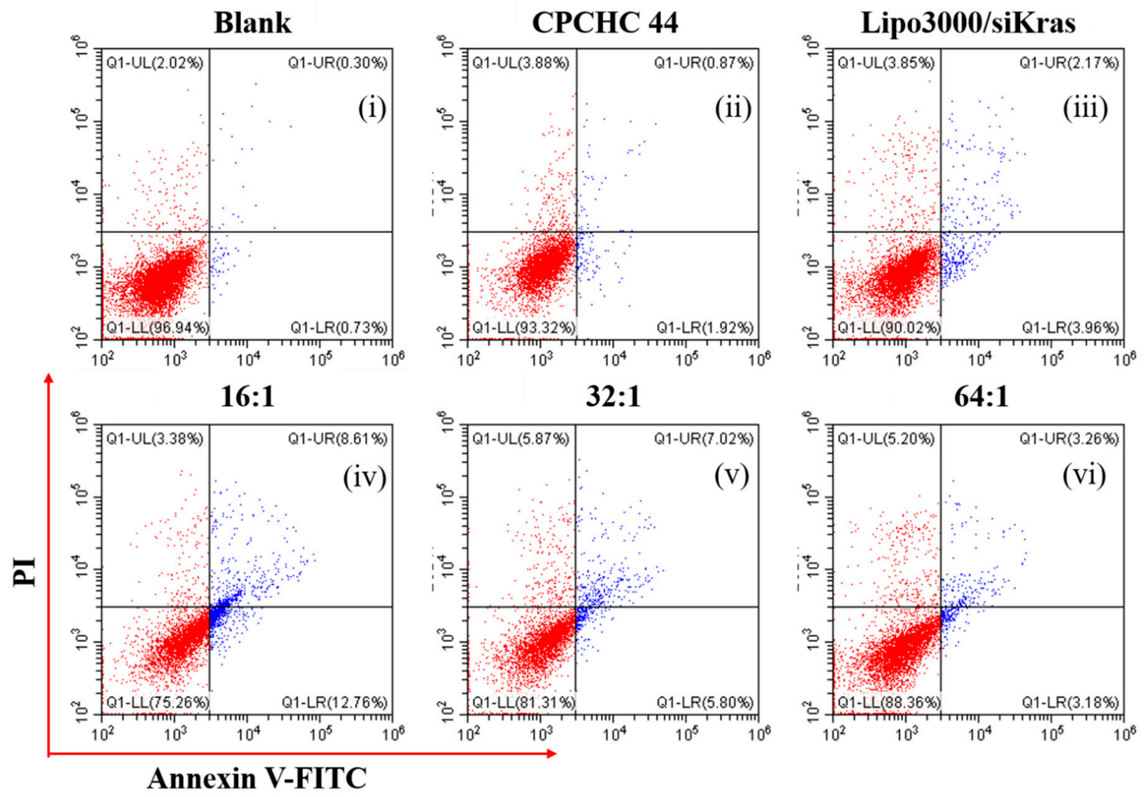

Figure S6. Flow cytometric analysis of cell apoptosis in PANC-1 and MiaPaCa-2 cells upon 72 h post-treatment treatments. The apoptotic cells were co-stained with annexin V and propidium iodide (PI). (a) Representative dot-pots of cells treated with (i) blank, (ii) CPCHC-44, (iii) Lipo3000/siKRAS, and different weight ratios of CPCHC-44/siRNA NPs: (iv) 16:1, (v) 32:1, (vi) 64:1.

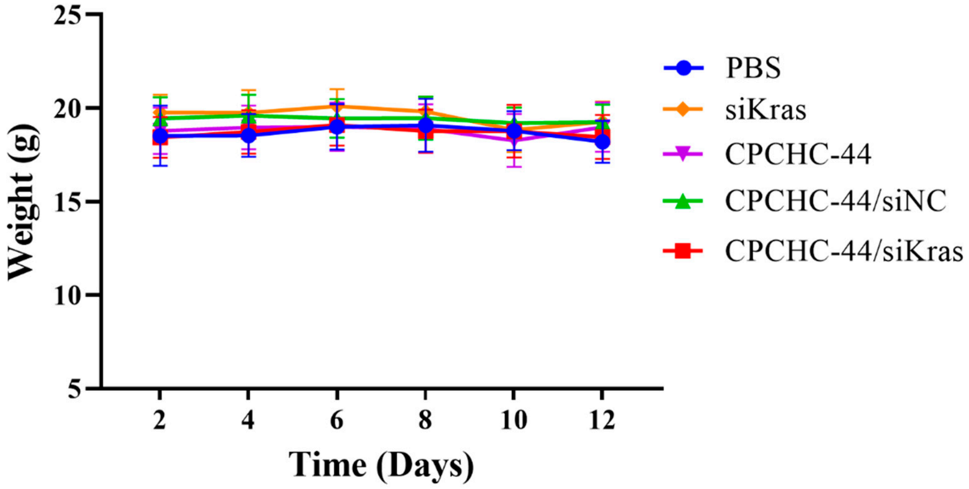

Figure S7. The body weight change profiles of different CPCHC 44-based formulations treatment.

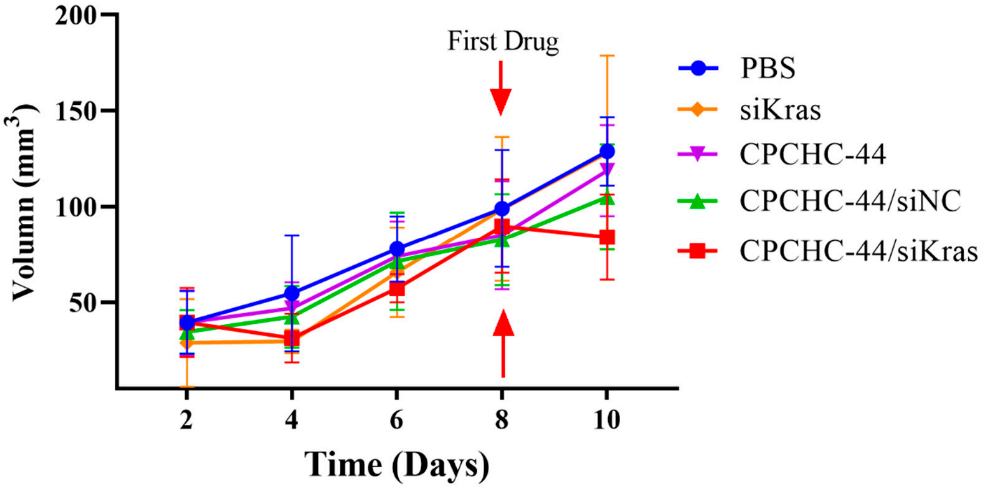

Figure S8. Tumor growth curves of different groups after different CPCHC 44-based formulations treatment. Tumor volumes have been normalized to the initial sizes.
